# Urbanization in China drives soil acidification of *Pinus massoniana* forests

**DOI:** 10.1038/srep13512

**Published:** 2015-09-24

**Authors:** Juan Huang, Wei Zhang, Jiangming Mo, Shizhong Wang, Juxiu Liu, Hao Chen

**Affiliations:** 1 Key Laboratory of Vegetation Restoration and Management of Degraded Ecosystems, South China Botanical Garden, Chinese Academy of Sciences, Guangzhou 510650, China; 2 Guangdong Provincial Key Laboratory of Environmental Pollution Control and Remediation Technology, Guangzhou 510275, China

## Abstract

Soil acidification instead of alkalization has become a new environmental issue caused by urbanization. However, it remains unclear the characters and main contributors of this acidification. We investigated the effects of an urbanization gradient on soil acidity of *Pinus massoniana* forests in Pearl River Delta, South China. The soil pH of pine forests at 20-cm depth had significantly positive linear correlations with the distance from the urban core of Guangzhou. Soil pH reduced by 0.44 unit at the 0–10 cm layer in urbanized areas compared to that in non-urbanized areas. Nitrogen deposition, mean annual temperature and mean annual precipitation were key factors influencing soil acidification based on a principal component analysis. Nitrogen deposition showed significant linear relationships with soil pH at the 0–10 cm (for ammonium N (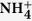-N), *P* < 0.05; for nitrate N (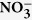-N), *P* < 0.01) and 10–20 cm (for 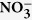-N, *P* < 0.05) layers. However, there was no significant loss of exchangeable non-acidic cations along the urbanization gradient, instead their levels were higher in urban than in urban/suburban area at the 0–10 cm layer. Our results suggested N deposition particularly under the climate of high temperature and rainfall, greatly contributed to a significant soil acidification occurred in the urbanized environment.

Soil acidification has been recognized as a serious environmental issue, especially in tropical regions, it declines terrestrial biodiversity, fine root biomass, and plant growth and productivity^1^,^2^,^3^,^4^, and threatens ecosystem health^5^,^6^,^7^. Continuous N deposition accelerates soil acidification in the tropical and temperate regions, resulting in adverse situations such as net decrease in soil pH and leaching of base cations, etc^8^,^9^,^10^,^11^.

At present, urbanization is an important outcome of development worldwide, especially in developing countries^12^, it is characterized by high densities of population and industry. During the process of urbanization, accumulation of numerous acidic and non-acidic pollutants, including N-and S-containing pollutants and metals, occurs. Urbanization, as an important source of N pollution, greatly affects N deposition by changing its compositions and sources^9^. Moreover, N deposition is higher in urban than in rural areas^13^,^14^. Therefore, urbanization might have a potential contribution to soil acidification.

Previous studies have indicated that urbanization alkalized soil because of the inputs of some alkaline ions, e.g. calcium (Ca^2+^) or sodium (Na^+^) ions, by investigating cases^15^,^16^,^17^ with higher or more alkaline pH values in the urban environment^18^,^19^. Hence, soil pH decline along the rural-to-urban gradient reported in some studies has not been paid adequate attention. For example, Pouyat *et al*.^20^ found that soil pH varied from 4.38 in urban stands to 4.61 in rural stands in New York. Further, they reported a decrease of soil pH in three metropolitan areas along the rural-to-urban gradient^17^. In addition, they^17^ explained that the decrease in pH was not very significant and could be overlooked, because acidic deposition could be neutralized by the enhanced inputs of alkaline ions. However, several studies from China have suggested the occurrence of soil acidification in urbanized regions, including Yangtze River Delta (YRD)^21^,^22^ and Pearl River Delta (PRD)^22^,^23^,^24^. Moreover, Zhang *et al*.^22^ showed that soil acidification in PRD was more severe than that in YRD, and Hou *et al*.^23^ indicated the risk of Al^3+^ and acidity stress was higher in the remnant forests in the PRD. Thus, soil acidification caused by urbanization has become a new environmental issue and should receive special attention. However, very little is known about soil acidification and its main drivers in urbanized environments.

Since 1978, urbanization has progressed rapidly in China because of the “open door policy” and economic reform. The PRD region is one of the three economic zones in China, and has shown the fastest urbanization in the world over the past 20 years^25^. China, especially PRD located in the tropical region, has become an important area of research on soil environment affected by urbanization according to published data^26^,^27^. In this study, fourteen Masson pine (*Pinus massoniana* L.) plantations along an urban-to-rural gradient in the PRD, South China, were selected to investigate the effects of urbanization on soil acidification. We hypothesized that (1) the urbanization gradient based on the distance from the urban core could show the trend of soil acidification of pine forests; (2) inorganic N deposition, especially 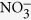-N deposition, significantly contributes to this acidification; and (3) base cations does not cause significant leaching along the pH decline.

## Results

### Soil pH decline along the urbanization gradient

**Figure 1:**
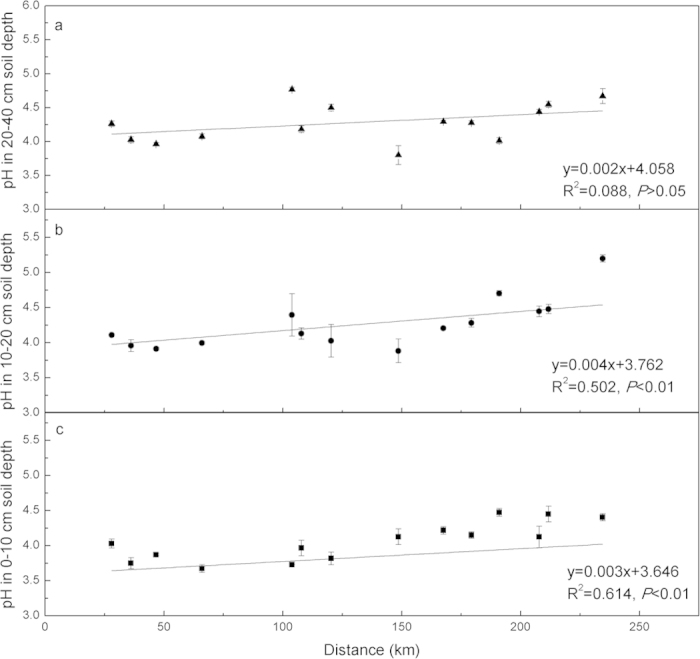
Linear relationship between soil pH values and the distance from urban core of Guangzhou city in Guangdong Province. (**a**), 20–40 cm soil depth; (**b**), 10–20 cm soil depth; (**c**), 0–10 cm soil depth. The results of linear regression analyses and the significance levels (*P*) are shown. In all case, best fit was obtained by linear regression (*y* = *a* + *bx*) analysis. *Error bars* indicate ±1S.E. (*N* = 3).

Soils in the studied pine forest were mostly acidic, with pH values ranging from 3.6 to 5.3. The pH values varied depending on soil depth and the distance from the urban core; the lowest soil pH was at the 0–10 cm depth, followed by that at the 10–20 cm and 20–40 cm depths (Fig. 1). A significant linear relationship between pH values and distance from the urban core was observed both at the 0–10 cm (R^2^ = 0.614, *P* < 0.01) and 10–20 cm (R^2^ = 0.502, *P* < 0.01) depth, but not at the 20–40 cm (R^2^ = 0.088, *P* > 0.05), suggesting that soil acidification was significantly higher along the distance from the urban core at the 20-cm depth.

The average pH values across the four gradients (urban areas, urban/suburban areas, suburban/rural areas, rural areas) classified according to distance from the urban core were also calculated; an increasing pH trend from urban to rural areas was noted. At topsoil (0–10 cm), pH was significantly different between urbanized regions (urban and urban/suburban sites) and non-urbanized (suburban/rural and rural sites) regions, and the average value reduced by 0.44 unit in the urbanized sites compared to that in the non-urbanized sites.

### Changes in exchangeable cations concentrations

**Figure 2:**
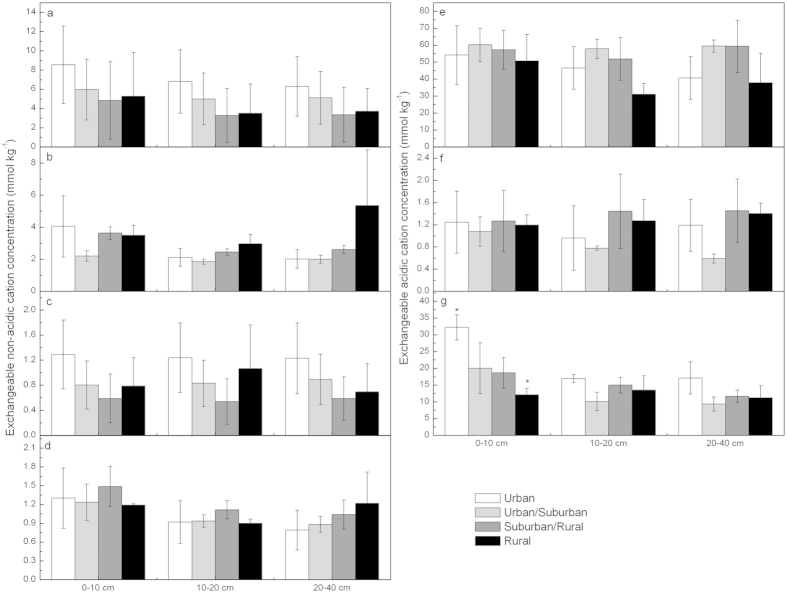
Contents of exchangeable cations among four urbanization gradients. (**a**), soil exchangeable Mg^2+^; (**b**), soil exchangeable Ca^2+^; (**c**), soil exchangeable Na^+^; (**d**), soil exchangeable K^+^ (**e**), soil exchangeable Al^3+^; (**f**), soil exchangeable Fe^3+^; and (**g**), soil exchangeable H^+^. *Error bars* indicate ±1S.E. (*N* = 3 for urban and rural, *N* = 4 for urban/suburban and suburban/rural). Asterisks (*) indicates significant differences at *P* < 0.05 levels between gradient classes.

In the PRD region, the concentrations of soil exchangeable cations decreased significantly with depth. For a given soil layer, no significant differences were observed in soil exchangeable cations and cation exchange capacity (CEC) among the four urbanized areas, except that H^+^ was significantly higher in urban than in rural areas at the 0–10 cm (*P* < 0.05). The levels of exchangeable cations except that of Al^3+^ remained higher in the urban areas than in the urban/suburban areas, especially at the 0–10 cm depth, although there were no significant differences (Fig. 2).

**Figure 3:**
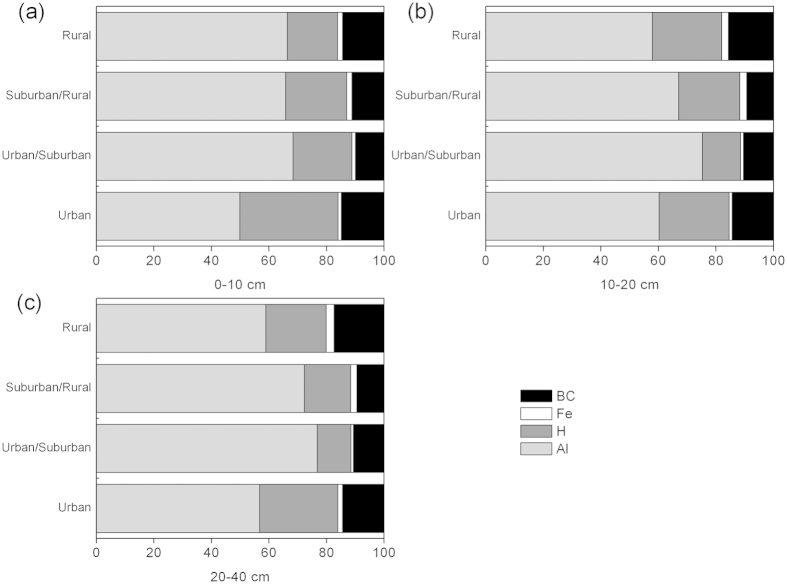
Effects of urbanization on relative composition of soil exchangeable cations (percentage charge of total exchangeable cations) in 0–10 cm (a), 10–20 cm (b), 20–40 cm (c) soils. ***Note***:BC, the total base cations of K^+^, Na^+^, Ca^2+^, and Mg^2+^.

Aluminum ion (Al^3+^) and H^+^ accounted for more than 80% of total exchangeable cations, with the dominant component being Al^3+^, comprising approximately 50%–78% of the total. Base cations (K^+^, Na^+^, Ca^2+^, and Mg^2+^) accounted for less than 15% of the total exchangeable cations, and Fe^3+^ accounted for less than 1% and 3% of the total at the 0–10 cm and 10–40 cm depths, respectively (Fig. 3). Magnesium ion (Mg^2+^) was the most abundant base cations, followed by Ca^2+^, K^+^ and Na^+^ (Fig. 2). Moreover, the concentrations of Mg^2+^ were significantly correlated with those of Na^+^ (r = 0.979, *P* < 0.01).

### Key factors influencing soil acidification

**Figure 4:**
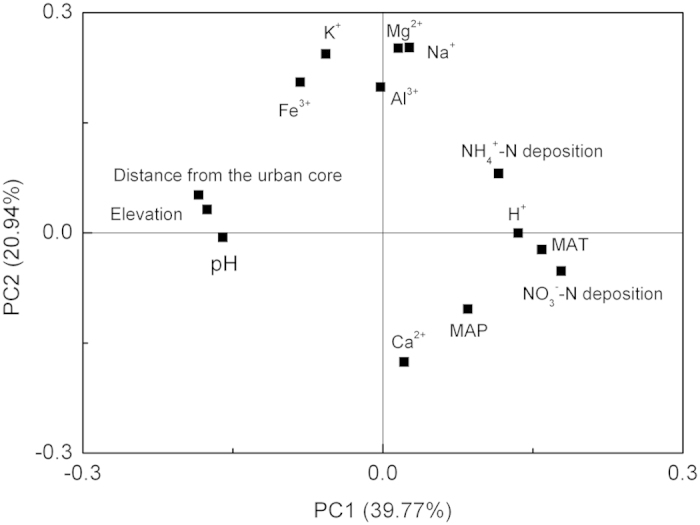
Principal component analysis (PCA) of the environmental factors, N deposition and soil pH and exchangeable cations at 0–10 cm of pine forests in the PRD region. MAT, mean annual temperature; MAP, mean annual precipitation.

To find the keystone parameters of soil acidification of pine forests in PRD, principal component analysis (PCA) was performed using all soil exchangeable cations, pH, N deposition and environmental factors (Fig. 4). Three principal components were observed in the PRD region (Fig. 4), which can contribute to 72.58% of this acidification. The first principal component included soil pH, distance from the urban core and elevation, the second principal component included 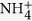-N and 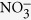-N deposition, mean annual temperature (MAT), mean annual precipitation (MAP), exchangeable H^+^ and Ca^2+^, and the third principal component included exchangeable K^+^, Na^+^, Mg^2+^, Al^3+^ and Fe^3+^. Therein N deposition as an environmental pollutant was an important factor for soil acidification, and MAT and MAP were also largely responsible for this change.

### Relationships between atmospheric N deposition with soil pH and fine root biomass

**Figure 5:**
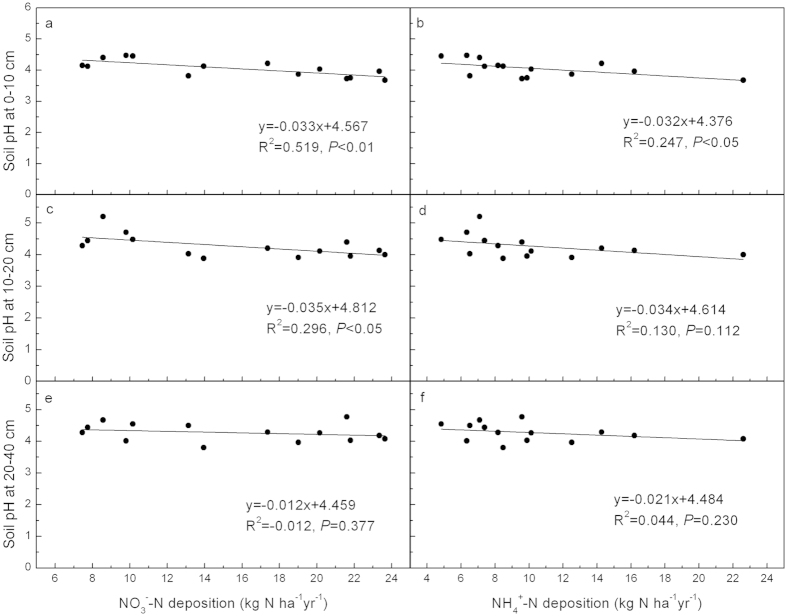
Linear relationship between soil pH with atmospheric inorganic N deposition in the PRD region, China. (**a**) and (**b**), linear between soil pH at 0–10 cm soil depth with 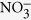 -N and with 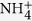-N deposition; (**c**) and (**d**), linear between soil pH at 10–20 cm soil depth with 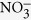 -N and with 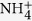-N deposition; (**e**) and (**f**), linear between soil pH at 20–40 cm soil depth with 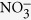 -N and with 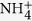-N deposition. The results of linear regression analyses and the significance levels (*P*) are shown. In all case, best fit was obtained by linear regression (*y* = *a* + *bx*) analysis.

Ammonium N (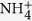-N) and nitrate N (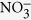-N) deposition had a significant linear relationship with soil pH at the 0–10 cm depth (*P* < 0.05), the latter showed a similar linear relationship even at the 10–20 cm depth (*P* < 0.05; Fig. 5), indicating that N deposition remarkably contributed to soil acidification in the PRD region.

A significant negative correlation was observed between live fine root biomass (data cited from Ref. 28) and 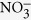-N deposition (r = −0.542, *P* < 0.05), but not with 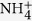-N deposition (r = −0.451, *P* > 0.05), suggesting that 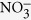-N deposition greatly inhibited the growth of fine roots.

## Discussion

### Significant soil acidification happening along the urbanization gradient

The soil pH remarkably declined along the urbanization gradient, since a linear relationship of pH with distance from the urban core was noted (Fig. 1). The pH is generally used as an indicator of soil acidity and it governs many ecologically important reactions^10^,^29^. The changes in soil pH generally require decades and even centuries before any appreciable changes in soil chemical characteristics can be observed^30^. Therefore, measurement of soil pH-associated changes over a short time is not possible^31^. Therefore, the decline of soil pH is considered a key feature of soil acidification^10^,^29^,^32^. Soil acidification occurred not only at the 0–10 cm but also 10–20 cm soil depth based on its significant decline along the distance from the urban core (Fig. 1). Our results revealed that urbanization could result in soil acidification rather than alkalinization, and soil acidification in urbanized regions (e.g., the PRD region) could have a significant impact at the 20 cm depth. Subsurface (10–20 cm) soil acidification mainly results from acid production of plant roots via the uptake of excess cations^33^,^34^. More rainfall with a mean annual precipitation of >1,500 mm in PRD also promotes soil acidification^35^,^36^ by leaching of nitrate and cations and retention of H^+^^33^,^34^. Consequently, more acid from the subsurface soil enters the topsoil^37^ and further accelerates its acidification.

Moreover, soil pH at the 0–10 cm layer was reduced by 0.44 unit in the urbanized areas (i.e. urban and urban/suburban areas) than in the non-urbanized areas (i.e. suburban/rural and rural areas). The magnitude of the decrease in soil pH of pine forest (0.44) was within the range of pH change of terrestrial ecosystem (0.08–0.49) caused by N deposition as revealed by a global analysis^36^, however, the magnitude was lower than the decrease of 0.50 reported in Chinese agricultural systems^32^ and of 0.63 reported in Northern China’s grassland^29^.

### Nitrogen deposition greatly contributes to soil acidification

The contribution of N deposition to soil acidification has been paid considerable attention because of its high level and tendency to increase annually^38^. Nitrogen deposition is known to cause soil acidification at the regional and global scale^29^,^32^,^36^,^39^. In the PRD region, N deposition exceeded 15 kg ha^−1^yr^−1^ in the four urbanization classes and surpassed 30 kg ha^−1^yr^−1^ in the urban/suburban and urban zones^13^, which is considered as one of the major contributors to soil acidification^11^,^38^. The contribution of atmospheric N deposition to soil acidification was supported by the significant correlations between atmospheric inorganic N deposition and soil pH at the 0–10 cm and 10–20 cm depths (Fig. 5) and the results from PCA (Fig. 4), which indicated that atmospheric N deposition contributes to soil acidification^40^,^41^. Similar linear relationship between soil pH changes of forests and N deposition was also reported by Yang *et al*.^39^.

In this study, 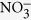 leaching driven by N deposition might be an important mechanism of soil acidification. This might occur as follow: (1) high N deposition will be noted in areas with a high risk of 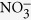 leaching^38^, and (2) conifer forests receiving high N deposition will exhibit higher 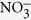 loss^42^. Nitrogen deposition (including 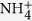-N and 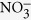-N) has important implications in terms of contribution to 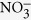 leaching, but enhanced 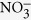 leaching is associated primarily with 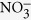-N deposition (including 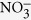 and oxidized N (NOx); Rothwell *et al*.^43^ and Curtis *et al*.^44^). Nitrate ion (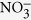) leaching from soil increases, and 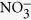 might become the dominant excess acid anion in the long term^45^, which was supported by the high level of 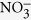 in the soil (data unpublished). In the PRD region, 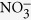-N was the main component of N deposition and mostly originated from NOx^13^. Moreover, NOx in the air can be oxidized to HONO and HNO_3_ via a photochemical reaction^46^. Therefore, 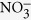-N deposition had greater contribution to soil acidification than 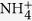-N deposition. The significant correlation between 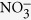-N deposition and soil pH (Fig. 5) also indicated that 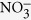-N deposition played an important role in soil acidification.

High N deposition could be responsible for the reduced fine root biomass^47^,^48^,^49^ based on the significant negative correlation between 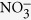-N deposition and live fine root biomass (r = −0.542, *P* < 0.05). Further, it could cause Al^36^ and Fe^3+^ release^8^,^38^, which contribute to higher Fe^3+^ in urban than in urban/suburban areas (Figs 2 and 3) besides the inputs from cities^16^,^17^.

Additionally, high temperature with a MAT of >19.4 °C and more rainfall with a MAP of >1,500 mm in this region (Table 1) promoted this soil acidification^35^,^36^ and their roles were supported by the results from PCA (Fig. 4).

### Features of soil acidification in the urbanized environment

Unlike in non-urbanized environment, two special soil features were observed in the urbanized environments. First, soil pH declined with the distance from the urban core according to significant linear relationships between soil pH at the 20-cm depth with the distance from the urban core (Fig. 2). Distance from the urban core was considered as an indicator of soil acidity changes in response to urbanization. Further, the indicative function of distance was supported by the significant negative correlations with atmospheric inorganic N deposition^13^ and soil organic carbon^28^ in PRD and with soil chemical properties^17^ and concentration and fluxes of 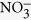, 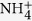, Ca^2+^, Mg^2+^, SO_4_^2−^, and Cl^−^ in throughfall^15^ in other regions. Second, there was no significant leaching of exchangeable base cations, instead, their levels were higher in the urban than the in urban/suburban areas (Fig. 2). The first reason could be greatly attributed to the important urban sources, e.g. coal combustion and building materials industries^16^,^17^. Sea salt^50^,^51^,^52^,^53^ from South China Sea was another factor responsible for the increase in the level of these cations (e.g. Na^+^, Mg^2+^ and K^+^). Calcium ion (Ca^2+^) can also be transported from long ranges^51^. Many inputs from urban areas compensate for the loss of base cations (e.g. Ca^2+^ and K^+^)^54^,^55^,^56^, resulting in higher levels of these cations at the 0–10 cm layer in the urban than in the urban/suburban (Fig. 2). Extra inputs of base cations could also contribute to the less proportion of Al to the total exchangeable cations in the urban areas compared to those in the urban/suburban areas (Fig. 3). The second reason was that the long-term effect (>5 years) of N deposition diminished the availability of exchangeable base cations^35^. In the PRD region, N deposition has increased since 1978 and has remained at a very high level, thus its negative effects on the loss of base cation might have greatly reduced.

## Conclusions

Remarkable soil acidification at the 20-cm cm depth occurred along the urbanization gradient at the PRD region. This acidification greatly decreased soil pH by 0.44 unit at the 0–10 cm depth in urbanized areas compared to that in non-urbanized areas. In this acidified soil, the levels of exchangeable non-acidic cations were maintained at higher concentrations in urban areas than in urban/suburban areas instead of leaching, because of the non-acidic cation sources available in urban areas. Nitrogen deposition, especially 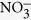-N deposition had a significant contribution to this acidification based on its impacts on soil pH, H^+^, fine root biomass, and soil exchangeable cation levels. High temperature and more rainfall in this region also promote this acidification. Therefore, controlling N pollutants, especially NOx, will be the first strategy for environmental management in China.

## Methods

### Study region and experimental design

The study area is located throughout Guangdong Province, south China. In this region, its environment gradients were observed from urban to rural sites: (1) annual average precipitation is also higher in urban areas than in rural areas^57^, (2) the total emission of anthropogenic NH_3_ reached 582.9 kt in 2010^58^, and (3) the number of motor vehicles in this region was more than 9.1 million in 2011^59^.

Four urbanization gradients in the range of 260 km, including urban, urban/suburban, suburban/rural, and rural, were classified based on the distance from urban core of Guangzhou City^9^,^28^. We divided each class into 10 subzones in equal areas. In each class we chosen 3 or 4 subzones to locate our studied forests at semi-random based on the land-use map. Fourteen pine plantations were chosen for the study. among them, three were in the urban class (Huolushan, Maofengshan and Shunfengshan, abbreviated to HLS, MFS and SFS, respectively), four in the urban/suburban class (Heshan - HS, Dinghushan - DHS, Guangyinshan - GYS, and Xiangtoushan - XTS), four in the suburban/rural class (Heishiding - HSD, Shimentai - SMT, Yunjishan – YJS, and Dachouding, DCD), and three in the rural class (Huaiji - HJ, Dadongshan - DDS, and Wuzhishan - WZS) (location of study sites see Chen *et al*., 2013b). The study regions have a warm and humid climate with annual precipitation ranging from 1566 to 2133 mm and mean annual air temperature from 19.65 to 22.22 °C. Their longitudes range from E111°54′19.78″ to E115′21′54.52″, and their latitudes from N22°46′0.60″ to N24°46′40.25″ (Table 1).

Pine plantations were selected because of their wide distribution in South China, accounting for 45% of total plantation area in Guangdong Province^60^. In addition, Masson pine forests have relatively structural and spatial homogeneity, eliminating the confounding of other factors. More importantly, it is very vulnerable and sensitive to environmental changes^61^,^62^. The pine forest plots were screened according to the following three criteria: (1) no forest fires, insect infestations, logging and fertilization, (2) far away from the edge of forests to avoid edge effect with similar slope and orientation, (3) stand ages between 40 and 60 years, and their stand density between 600 and 800 trees ha^−1^, (4) soils of lateritic red earth (Ultisols in USDA soil taxonomy) (Table 1).

### Soil sampling and measurement

Soils sampling was conducted from January to May in 2011. In each plantation, three random subplots (5 m × 5 m) were selected to sample soil from three mineral soil layers (0–10 cm, 10–20 cm and 20–40 cm) using a 10 cm inside diameter corer. Soil samples were passed through a 2-mm sieve to remove roots and stones, mixed thoroughly by hand. Soil samples were air-dried and used to determine pH and exchangeable cations. Soil pH was measured in a 5 g soil: 25 ml water suspension^63^. Exchangeable non-acidic cations (i.e. K^+^, Ca^2+^, Na^+^ and Mg^2+^) were extracted with 1 mol L^−1^ NH_4_Ac, and one exchangeable acidic cation, i.e. Fe^3+^ were extracted with 0.1 mol L^−1^ HCl^63^, then these cations were determined by inductively coupled plasma optical emission spectrometer (Perkin Elmer, USA). Exchangeable Al^3+^ content was calculated as the difference between total exchangeable acidity and the exchangeable H^+^ content. Exchangeable acidity (exchangeable H^+^ and exchangeable Al^3+^) was extracted with 1 M KCl using a 5 g soil: 500 ml solution. Half of the extract was titrated with 0.02 M NaOH solution to determine total exchangeable acidity, and the others were titrated with 0.02 M NaOH after adding 1 M NaF to obtain exchangeable H^+^ content^63^. Cation exchange capacity (CEC) was calculated as the sum of the charge equivalents of the exchangeable cations^23^.

### Atmospheric N deposition determination

The ion-exchange resin (IER) columns were used to quantify inorganic N deposition in bulk precipitation at the study sites^13^. A funnel was installed on the top of the IER column (a 16 mm × 330 mm polyvinylchloride (PVC) tube) with a septum and a fitting. A fine mesh screen was placed on the surface of the funnel to keep out debris. The resin used for IER collector is a mixture of strong base styrene anion-exchange resin (201 9 7[717], similar to Amberlite IRA-400) and strong acid styrene cation-exchange resin (001 9 7[732], similar to Amberlite IR-120; Guangzhou, China). 40 g of mixed resin (half cation and half anion) was added to each PVC column and rinsed with distilled water. At each plot, three to five IER columns were installed to collect precipitation, and two IER columns with both ends sealed were left in each site to determine background N contamination in the ion resin. The 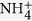-N concentrations were measured by the indophenol blue method followed by colorimetry, and 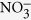-N concentrations were measured after cadmium reduction to 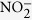-N, followed by sulfanilamide-nicotinamide adenine dinucleotide (NAD) reaction^63^. Wet inorganic N deposition was calculated using the method adopted by Sheng *et al*.^64^.

### Statistical analysis

Data of atmospheric inorganic N deposition (including 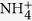-N and 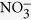-N deposition) and fine root biomass derive from Huang *et al*.^13^ and Chen *et al*.^28^, respectively. One-way analysis of variance (ANOVA) was used to compare the differences among four urbanization classes (urban, urban/suburban, suburban/rural, and rural) in soil pH, exchangeable cations and CEC. Linear regressions of soil pH to distance from the urban core and N deposition were determined to generalize the contribution of the urbanization to soil pH. Pearson correlation analysis was also performed to examine the relationships between soil pH and N deposition with, exchangeable cations and with fine root biomass. Principal Components Analysis (PCA) was used to generalize the effects on soil acidification from environmental factors (including elevation, mean annual temperature and mean annual precipitation), soil pH and exchangeable cations and N deposition. All analyses were conducted using SPSS 13.0 for windows, with statistical significant difference set with *P* value <0.05, unless otherwise stated. Mean values are expressed ±1 standard error of the mean.

## Additional Information

**How to cite this article**: Huang, J. *et al*. Urbanization in China drives soil acidification of *Pinus massoniana* forests. *Sci. Rep*. **5**, 13512; doi: 10.1038/srep13512 (2015).

## Figures and Tables

**Table 1:** Site characteristics.

Site(code)	Latitude(N)	Longitude(E)	Distancefrom urbancore (km)	Elevation(m a.s.l.)	MAP(mm)	MAT (°C)	Standdensity(trees ha^−1^)	Tree age(year)
HL	23°10′53.30″	113°23′2.00″	36.1	45	1742(351)	22.09(0.52)	700	40
MFS	23°18′5.87″	113°27′0.57″	46.7	50	1742(351)	22.09(0.52)	700	40
SFS	22°49′7.65″	113°16′38.99″	28.0	48	1742(351)	22.09(0.52)	700	50
HS	22°40′13.31″	112°54′14.01″	66.0	60	1701(283)	21.15(0.43)	700	40
DHS	23°8′57.27″	112°31′3.07″	107.8	283	1625(275)	22.22(0.47)	800	60
GYS	23°58′9.34″	113°33′49.22″	120.3	385	2133(383)	20.95(0.41)	700	50
XTS	23°18′26.87″	114°25′37.54″	103.8	366	1730(340)	22.01(0.49)	700	40
HSD	23°27′42.85″	111°54′19.78″	179.3	400	1690(265)	20.99(0.47)	700	50
SMT	24°23′7.47″	113°18′8.49″	167.5	56	1675(243)	19.45(0.43)	700	40
YJS	24°4′55.65″	114°10′18.33″	148.6	462	1758(314)	19.93(0.50)	700	40
DCD	24°16′58.67″	112°25′25.81″	191.0	891	1597(328)	19.65(0.45)	700	40
HJ	24°4′7.45″	111°57′50.40″	207.8	432	1597(328)	19.65(0.45)	700	40
WZS	24°46′40.25″	113°15′28.59″	211.7	500	1566(281)	20.38(0.39)	750	60
DDS	24°46′17.29″	112°30′3.17″	234.5	815	1597(328)	19.65(0.45)	700	60

MAP, long-term mean annual precipitation; MAT, long-term mean annual temperature. Temperature and precipitation interpolated from nearest meteorological station data. Latitude, longitude and elevation are from GPS readings taken on site.
